# The Coordination of mTOR Signaling and Non-Coding RNA in Regulating Epileptic Neuroinflammation

**DOI:** 10.3389/fimmu.2022.924642

**Published:** 2022-07-11

**Authors:** Chudai Zeng, Jason Hu, Fenghua Chen, Tianxiang Huang, Longbo Zhang

**Affiliations:** ^1^ Departments of Neurosurgery, and National Clinical Research Center of Geriatric Disorders, Xiangya Hospital, Central South University, Changsha, China; ^2^ Department of Neonatology, Yale School of Medicine, New Haven, CT, United States; ^3^ Department of Neurosurgery, Yale School of Medicine, New Haven, CT, United States

**Keywords:** epilepsy, neuroinflammation, mTOR, non-coding RNA, neural damage

## Abstract

Epilepsy accounts for a significant proportion of the burden of neurological disorders. Neuroinflammation acting as the inflammatory response to epileptic seizures is characterized by aberrant regulation of inflammatory cells and molecules, and has been regarded as a key process in epilepsy where mTOR signaling serves as a pivotal modulator. Meanwhile, accumulating evidence has revealed that non-coding RNAs (ncRNAs) interfering with mTOR signaling are involved in neuroinflammation and therefore articipate in the development and progression of epilepsy. In this review, we highlight recent advances in the regulation of mTOR on neuroinflammatory cells and mediators, and feature the progresses of the interaction between ncRNAs and mTOR in epileptic neuroinflammation.

## Introduction

Epilepsy, characterized by recurrent spontaneous seizures, is one of the most common neurological diseases affecting over 50 million people worldwide (1). Epileptic seizures can be traced to a broad spectrum of factors such as brain abnormalities and trauma (2). Extremely complicated pathophysiological processes are involved in epilepsy in which aberrant regulation of inflammatory cells and molecules have emerged to act as important mediators of epileptogenesis and epileptic injury, which is considered as an epileptic factor independent of etiology (3–5). Epileptogenesis is commonly associated with persistent inflammatory in the brain microenvironment (3). Conversely, seizures contribute to a cascade of inflammatory events as well leading the activation of pro-inflammatory molecules, glial proliferation and phenotypic transformation, breakdown of the blood-brain barrier (BBB), and subsequential neural damages (6).

The mammalian target of the rapamycin signaling (mTOR), as the key regulator of protein synthesis and autophagy, plays critical roles in neurophysiological processes such as neural development and brain circuit formation, as well as the continuum of neurological disorders (7–12). Recently, dysregulation of mTOR signaling has been implicated in infection and inflammatory progresses and acts as an intriguing component of the complex mechanism involved in epileptic neuroinflammation (9, 13). Non-coding RNAs (ncRNAs), defined as the RNAs that are not translated into functional proteins, have been demonstrated to affect gene translation and interfere with signaling pathways in diverse biological contexts, such as neuronal disorders, cancer and immune responses (14–16). In addition, ncRNAs has been found to be involved in neuroinflammation and participate in the initiation and progression of epilepsy by evidence accumulated over the past decade (2, 17–20), which provide their potentials of acting as biomarkers and therapeutic targets. In this review, we provide an overview of the current understanding on the coordination of mTOR signaling and ncRNAs in the regulation of epileptic neuroinflammation, and its potential contribution to the development and progression of epileptic seizures.

## Overview of mTOR Signaling

mTOR was originally identified as the target of rapamycin that consists of the catalytic subunits of two distinct multiprotein complexes: mTOR complex 1 (mTORC1) and 2 (mTORC2). mTORC1 is composed of mTOR, regulatory associated protein of mammalian target of rapamycin (Raptor), mammalian lethal with Sec13 protein 8 (mLST8), DEP domain containing mTOR-interacting protein (DEPTOR), and proline-rich AKT substrate of 40 kD (PRAS40). mTORC1 balances anabolic and catabolic processes by the stimulation of biosynthetic processes that produce proteins, nucleotides, and lipids required for cell growth and proliferation, and suppression of autophagic pathway (21, 22). mTORC1 integrates both intracellular and extracellular signaling including growth factors, energy, oxygen, and amino acid levels (23), and regulates a broad spectrum of downstream effectors though S6 kinases (S6K) and eIF4E-binding protein 1 (4EBP1) (24, 25). mTORC2 is composed of mTOR, rapamycin-insensitive companion of mTOR (Rictor), mLST8, DEPTOR, and the regulatory subunits mSin1 and Protor1/2. mTOC2 senses insulin, growth factors, etc., and phosphorylate mainly AGC kinases. mTORC2 is essential for cell survival and the maintenance of the actin cytoskeleton (26, 27).

## mTOR and Epileptic Neuroinflammation

### Neuroinflammation in Epilepsy

Neuroinflammation as one of the most important pathophysiological traits of epilepsy is the inflammatory response to epileptic seizures within the brain. Although the aspects of neuroinflammation can vary due to the diverse types of seizure, the main context and course are shared. Epileptic neuroinflammation commonly presents abnormal activations of resident central nervous system (CNS) glia (microglia and astrocytes) and endothelial cells, recruits of peripherally derived immune cells *via* the disruption of BBB, and the increased releases of inflammatory cytokines, chemokines, reactive oxygen species, and secondary messengers. Moreover, epileptic neuroinflammation can lead to the consequences of edema, neural damage, or even cell death, and acts as a “second hit” to trigger or worsen epilepsy.

Microglia cells are regarded as resident macrophages of brain and are able to polarize to pro-inflammatory M1 or anti-inflammatory M2 phenotypes with distinct physiopathological functions. The hyperactivation of microglial is primarily found in epileptic neuroinflammation with releasing of high-mobility group box-1 (HMGB1), a pro-inflammatory mediator (28, 29). Glia cells and astrocytes also respond to inflammatory signals and crosstalk with other cells, such as microglia, neurons, endothelial cells, and peripheral immune cells (30, 31). Glia cells along with other cells in the brain such as neurons and endothelial cells express pro-inflammatory mediators during neuroinflammation in various neurological disorders. Reactive astrocytes triggered by seizures are important cellular components involved in both pro-inflammatory and anti-inflammatory processes in neuroinflammation (32–34). They can release a wide range of inflammatory cytokines and chemokines that complement cascade components (35). Overreactions of astrocytes have been reported to disturb synaptic activity and linked to neuronal loss in epileptic inflammation (36, 37).

BBB, an important structure consisting of capillary walls and astrocytes, is temporally and anatomically associated with epilepsy (38, 39). The disruption of BBB structure and permeability induced by neuroinflammation promotes peripheral immune cells and inflammatory mediators to enter the brain microenvironment. As a result, the infiltration of peripheral immune cells such as monocytes and T cells can impair BBB as well (40). Monocytes have been gradually studied as potential treatment targets for epilepsy. Monocytes can migrate *via* the bloodstream to the brain though disrupted BBB to sustain seizure activity and promote neuroinflammation by differentiation into macrophages and microglia-like cells (41–44). C-C motif chemokine ligand 2 (CCL2) and its receptor CCR2 are essential for the process (45). Lymphocytes that participate in the adaptive immune system are recruited to the brain in epileptic neuroinflammation as well (46–48). Pro-inflammatory CD4+ and CD8+ T cells have been observed to infiltrate the brain in seizure animals (49, 50). Interestingly, regulatory T cells (Tregs), a type of tissue-resident lymphocytes that participate in the negative regulation of immune responses, are less common in the normal brain but significantly accumulate in neuroinflammation (51).

Proinflammatory molecules, such as cytokines, chemokines, and growth factors, are important mediators in epileptic neuroinflammation, which regulate onset and development of seizures. In addition, the inflammatory mediators can cause secondary damage and contribute to repetitive seizures (4, 52–55). Proinflammatory Interleukin (IL)-1, released from glial cells and white cells, plays a crucial role in neuroinflammation (56). In seizure models, the concentration and activation are significantly increased, which enhance the neuronal excitability and thus exacerbate neuronal hyperactivity and excitotoxicity (34, 45, 57). Tumor necrosis factor alpha (TNF-α), a pro-inflammatory cytokine primarily released from microglia, has recently reported to be involved in the etiology of epilepsy and attenuate aberrant neurogenesis (4, 58, 59). Chemokines secreted from astrocytes, microglia, and endothelial cells contribute to seizures and epileptogenic progression (32, 57) and have been suggested to further recruit more inflammatory cells. Hyperexpression of CCL2 in epilepsy is suggested to increase seizure-induced IL-1β production and neuronal cell death (30, 45). Recent studies show that interference with CCL2 signaling effectively inhibits seizures (60). Danger-associated molecular products (DAMPs) such as HMGB1, complements, ATP, reactive oxygen species (ROS) are cell-derived molecules that are released during tissue damage. The endogenous signals are recognized by toll-like receptors (TLRs) that belong to innate immunity and are vital in neuroinflammation, and the receptor for advanced glycation end products (RAGE) as well (4, 61–65).

### mTOR and Inflammatory Cells

mTOR signaling is a critical regulator of the function of immune cells. Previous studies have demonstrated that mTORC1 signaling and the protein levels of inflammatory mediators in microglia are upregulated (66). Inhibition of mTOR decreased the formation of autophagosomes and production of lipopolysaccharides (LPS)-induced proinflammatory cytokines in microglia (67, 68). In neuroinflammation, mTOR can modulate microglia polarization and thus function though metabolic reprogramming (67, 69). Suppression of mTOR activity is suggested to attenuate the microglial activation and therefore alleviate seizure severity (70). However, another study indicated that mTOR-deficient microglia in status epilepticus reduces induction of inflammation and thus increases seizure susceptibility (71). In addition, mTOR activation could promote the growth of neurites by promoting the releases of astrocyte-derived neurotrophic factors (72). Inhibition of mTOR pathway by rapamycin mitigates astrocyte migration, proliferation, and production of inflammatory mediators (73).

In epileptic neuroinflammation, mTOR hyperactivation leads to the disruption of BBB facilitating the infiltration of peripheral immune cells (74, 75). Meanwhile, mTOR advocates the development of monocyte and macrophage in the bone marrow by reducing macrophage colony-stimulating factor receptor CD115 expression and stimulates monocytes to enter M2-like macrophages (76, 77). Interestingly, monocytes infiltration can upregulate microglia activation after seizure induction (45). mTOR also regulates differentiation and activation of T cells and T helper (Th) cells. mTOR inhibition decreases effector molecules in both CD4+ and CD8+ cells (78) and maintain the quiescence state of naïve cells (79, 80). mTORC1 has been reported to coordinate metabolic programs to selectively regulate Th1 and Th17 differentiation (81, 82). While mTORC2 promotes the differentiation of Th2 cells (81, 83). Interestingly, the inhibition of mTOR by rapamycin can promote the development of regulatory Treg cells from naive T cells and improve the quality of memory CD8+ T cells, mainly due to the enhancement of autophagy activity (84, 85).

### mTOR and Neuroinflammatory Mediators

#### Cytokines and Chemokines

mTOR regulates a number of inflammatory cytokines and chemokines expression in epileptic neuroinflammation. IL-1β, for example, has been shown to be upregulated in mesial temporal lobe epilepsy (MTLE). Inhibition of mTOR activity by rapamycin blocks secretion of the IL-1β though the activation of autophagy, which could block the differentiation of Th17 cells (86–88). IL-17, mainly derived from Th17 cells, is also increased though mTOR activation in epilepsy, which leads excessive pro-inflammatory cytokine expression and maintain the chronic inflammation (48, 89). mTOR can also modulates IL-17 expression indirectly *via* several pathways, including signal transducer and activator of transcription 3 (STAT3) and hypoxia-inducible factor (HIF)-1α, which is suggested to be induced by IL-1β (90–92). Of note,

IL-17 can activate mTOR pathway and inhibit autophagy conversely (93, 94). In addition, transforming growth factor-β (TGF-β) released by reactive astrocytes is considered as an epileptogenic factor, which can preserve cellular metabolism in T cells and maintain response and promote neuron autophagy in a mTOR-dependent manner in neuroinflammation (95–97). Moreover, mTOR has been demonstrated to stabilize TNF-α mRNA released from microglia (67).

#### Danger-Associated Molecular Products

DAMPs have been shown to initiate proinflammatory immune responses from nonneuronal glial cells and contribute to the chronic neuroinflammation present in seizures accelerating the degeneration of neurons. HMGB1, a representative DAMPs, is secreted by immune cells as a cytokine mediator of inflammation involved in epileptic neuroinflammation (98–101). mTOR acting as the downstream effector is suggested to be implicated in HMGB1-induced maturation and antigen-presenting ability of dendritic cells and secretion of proinflammatory cytokines (102). In addition, nitric oxide (NO) is a neurotransmitter that regulates the differentiation and proliferation of neurons, and is proven to be involved in the anti-seizure effects of morphine and thiamine (103–105). mTOR can promote the release of inducible nitric oxide synthase (iNOS) as well as iNOS mRNA stability in astrocytes (106, 107), which in turn can activate mTOR pathway by a reversible nitrosylation of tuberous sclerosis complex (TSC) 2 (108). Moreover, excessive ROS in neurons has been found to be associated with epileptic injury (109). mTOR serves as a modulator of ROS production as well as an important effector in response to ROS (67, 110, 111).

### mTOR-Targeted Epilpesy Therapy

Drugs targeting mTOR have been widely studied, especially for the treatment of refractory epilepsy, which present promising anti-seizure effects in both lab investigation and clinical trials. Recently, Everolimus, a rapamycin derivative, is approved by FDA for the treatment of tuberous sclerosis complex-associated partial-onset seizures. However, clinical evidence indicates that solo inhibition of mTOR activity isn’t fully effective to cure epilepsy and epilepsy associated pathophysiological changes, and can be associated with serious adverse events, such as protein synthesis disorder (112), suggesting other unknow molecular mechanisms are involved in epileptogenesis (113), and more precise targeting of specific molecules in mTOR pathway is required to reduce side effects.

## mTOR Related Non-Coding RNA

Non-coding RNAs (ncRNAs) consist of intronic RNAs, microRNAs (miRNAs), long noncoding RNAs (lncRNAs), circular RNAs (circRNAs), and extracellular RNAs (114). ncRNAs can exert biological effects *via* directly targeting mTOR, targeting other components of mTOR complex or targeting key upstream regulators of mTOR. Conversely, mTOR can also regulate ncRNA biogenesis. For example, Raptor, an essential component of mTORC1 controls miRNA biogenesis though Drosha, a RNase processing primary miRNA (pri-miRNA) to precursor miRNA (115). GSK3β, a downstream molecule of mTOR, directly increases Drosha activity and mature miRNAs accumulation (116). Studies also suggested mTOR regulation of miRNA expression through its upstream enhancer or transcription factor (117, 118). In epilepsy, ncRNAs can regulate inflammatory cells and mediators by interacting with mTOR signaling during the intricate neuroinflammatory processes. Here, we focus on the functions of three main categories of ncRNAs (miRNAs, lncRNAs, and circRNAs) on epileptic neuroinflammation, as well as the interrelation with mTOR signaling.

### miRNAs

miRNAs, a major class of functional noncoding RNAs, have emerged as important post-transcriptional regulators of gene expression and provided a completely new manner in manipulating gene expression (114). miRNAs are short noncoding RNAs (20-23 nucleotides) that recognize partially complementary target sequences in selected mRNAs, and predominantly inhibit protein expression by either destabilizing their mRNA targets or inhibiting protein translation. A single miRNA can have multiple targets and have diverse effects on gene translation, which forms a complex regulatory signaling network (119). Series of studies have demonstrated that miRNAs mediate neuroinflammation and play a crucial role in the development and progression of epilepsy *via* mTOR pathway (see **Table 1**).

**Table 1:** Summary of mTOR-related miRNAs in epileptic neuroinflammation.

microRNA	Expression level in epilepsy	Function on neuroinflammation	Directly Targeting mTOR	Reference
**miR-21**	up	upregulation	no	(120–122)
**miR-23**	up	downregulation	no	(123–125)
**miR-27a**	up	upregulation	no	(126, 127)
**miR-101**	controversial	downregulation	yes	(128–130)
**miR-124**	down	controversial	no	(131–134)
**miR-129**	up	downregulation	yes	(135–137)
**miR-132**	up	downregulation	no	(138, 139)
**miR-144**	controversial	downregulation	yes	(130, 140, 141)
**miR-146a**	up	downregulation	no	(142–145)
**miR-155**	up	upregulation	no	(146, 147)
**miR-221/222**	down	upregulation	no	(148, 149)

miR-21-5p is an inflammatory miRNA increased in epilepsy. Recent studies have suggested that miR-21-5p can modulate the proliferation and apoptosis of astrocytes in neuroinflammation by targeting phosphatase and tensin homolog (PTEN)-mTOR signaling. Inhibition of miR-21-5p has the potential of preventing neuronal damage in stages of epileptogenesis (120, 121, 150). mTOR, in turn, can enhance the expression of miR-21 though transcription factor STAT3, a direct substrate of mTOR (151, 152). Meanwhile, miR-21-5p has been reported to decrease in hippocampal sclerosis (HS) and focal cortical dysplasia (FCD) (122, 153). Further research on miR-21-5p would be appreciated. miR-23 has been reported to interact with mTOR signaling and contribute to temporal lobe epilepsy (TLE) (123, 124). miR-23a can reactivate the AKT/mTOR pathway and suppresses neuroinflammation (154), while miR-23b suppresses IL-17-associated autoimmune inflammation and alleviates brain injury by interacting with AKT/mTOR pathway on negative regulation of inositol polyphosphate multikinase (IPMK) in pediatric refractory epilepsy (48, 125, 155). miR-27 acting as a regulator of phosphoinositide 3-kinase (PI3K)/AKT/mTOR axis widely participates in metabolic and inflammatory processes (156–158). miR-27a has been shown to be upregulated in epilepsy patients and inhibition of miR-27a alleviates the inflammatory response (126). Recently, miR-27a has been suggested as a potential serum biomarker for diagnosis and mechanistic links to underlying pathomechanisms in adult TLE (127). In addition, miR-124 is one of the most abundant miRNA identified in the CNS (159), which has been reported controversially in the mediation of epilepsy. miR-124 presents the inhibition of susceptibility of epileptic seizures *via* regulating cAMP-response element-binding protein1 (CREB1) or neuron restrictive silencer factor (NRSF) (131, 132). However, other studies have shown that miR-124 can promote epilepsy *via* enhancing microglia activation and inflammatory cytokines (132). By interacting with mTOR, miR-124 conducts mostly negative regulations on neuroinflammation (133, 160). miR-124 derived from microglia promotes the anti-inflamed M2 polarization and inhibits neuroinflammation *via* the suppression of mTOR signaling (134). Moreover, miR-146a has been widely studied in metabolic dysfunction and neuroinflammatory response (142). miR-146a is persistently expressed in reactive astrocytes to protect the CNS from neuroinflammatory damage in epilepsy (143, 161). Interestingly, miR-146a are upregulated commonly associated with IL-1β in epilepsy (162). A possible explanation is that enhanced miR-146a can upregulate IL-β by downregulating complement factor H (CFH), and facilitates miR-146a-CFH-IL-1β feedback loop that maintains chronic inflammation (144). miR-146a can suppress apoptosis and thus regulate the function of macrophage in neuroinflammation through mTOR pathway (163, 164), while mTORC1 can reactivate miR-146 micro-ribonucleoproteins (miRNPs) to restrict proinflammatory cytokine production in amyloid beta-exposed glial cells (165). In addition, miR-146a has been observed in the serum of both epilepsy patients and animal models (145, 166), offering the potentials of biomarker for both diagnosis and prognosis. Finally, miR-155 tag single-nucleotide polymorphisms (SNPs) are genetic susceptibility factors for epilepsy, where miR-155 may positively participate in the neuroinflammatory process (146, 167). For example, under the exposition of LPS, microglia increase the releases of nitric oxide and inflammatory cytokines by upregulating miR-155 (168). mTOR signaling and miR-155 could be directly targeted to each other. mTOR related epileptic disorders, such as TSC, display high expression of miR-155 and oxidative stress markers (169), while miR-155 can regulate mTOR activation controlling estrogen receptor function (147).

Additional microRNAs have been revealed to be involved in epileptic neuroinflammation with the interaction with mTOR signaling. Altered expression of miR-144 has been reported in TLE (170), which may modulate endothelial cells and macrophages in neuroinflammation by directly interacting with the 3’ untranslated regions (UTRs) of mTOR (140, 141, 171, 172). Astrocyte-derived miR-221/222 signals mTOR pathway leading the regulation of immune responses and autophagy through cytokines and cell adhesion molecules in epileptic neuroinflammation (148, 149). Moreover, miR-132 increases in astrocytes in TLE and promotes the activations of molecules in PI3K/mTOR pathway, such as AKT, TSC2, mTOR (138, 173, 174), which decrease the expression of inflammatory mediators including IL-1β、TGF-β、CCL2 (139).

### LncRNA

LncRNAs are large and heterogeneous ncRNAs with more than 200 nucleotides in length, which regulate gene expression at transcriptional, post-transcriptional, chromatin remodeling and epigenetic levels and act as competing endogenous RNAs (ceRNAs) with miRNAs (175, 176). Diverse functions of lncRNAs have been identified in epileptic neuroinflammation.

#### Pro-Neuroinflammatory lncRNAs

LncRNA ILF3-AS1 is a newly discovered lncRNA in epilepsy which promotes the expression of inflammatory cytokines by targeting miR-212 in TLE (177). However, upregulation of ILF3-AS1 could have a protective effect of alleviating hypoxic injury *via* activation of PI3K/AKT signaling pathway (178). ZFAS1 has been reported to be upregulated in epilepsy which promotes neuronal apoptosis and neuroinflammation (179). The inhibition of PI3K/AKT pathway can silence ZFAS1 leading the downregulation of epilepticus-induced neural autophagy (180). TUG1 positively regulates inflammatory processes in many neurological diseases such as epilepsy, cognitive impairment, and ischemic stroke (181, 182). In epilepticus, downregulation of TUG1 relieves neuronal apoptosis and the releases of inflammatory cytokines *via* targeting the miR-421/mTOR and miR-199a-3p/mTOR axes (183–185). The nuclear paraspeckle assembly transcript 1 (NEAT1) has been demonstrated to regulate both neural activity and inflammation though interacting with mTOR pathway in epilepsy (186, 187). It binds epilepsy-associated potassium channel-interacting proteins and subsequently induces neuronal hyperexcitation contributing seizure initiation (188). Meanwhile, NEAT1 promotes IL-6, cyclooxygenase (COX)-2, and TNF-α expression by targeting miR-129-5p and activating the Notch signaling pathway in epilepsy (136). In addition, excessive NEAT1 can impair the integrity and augments the permeability of BBB, worsening neuroinflammation (189). Moreover, H19 has been found to activate astrocytes and microglia, and prompt inflammatory responses and neuron apoptosis in epileptic seizures *via* the binding and suppression of miRNA let-7b and blocking mTORC1-mediated 4EBP1 phosphorylation (190–193).

#### Anti-Neuroinflammatory lncRNAs

Brain-derived neurotrophic factor (BDNF) antisense RNA (BDNF-AS) acts a natural antisense lncRNA of BDNF, a canonical nerve growth factor to support the survival of neuronal population and has been suggested as a potential target of epilepsy (194, 195). The regulation of BDNF-AS on seizure activities probably attribute to its activation of mTOR by inhibiting ribonuclease inhibitor 1 (RNH1)-mediated mTOR mRNA decay, as well as the controlling on the production of BDNF related inflammatory cytokines, such as TNF-α, IL-2, IL-6 (196, 197) and MALAT1, participates in the anti-inflammatory processes and regulates autophagy in endothelial cells and neurons by interacting with mTOR pathway in epilepsy (198–201). MALAT1 can induce splicing factor Ser/Arg-rich splicing factor 1 (SRSF1) and thus modulates the alternative splicing of S6K1 to activate mTOR signaling (202). It is also noteworthy that MALAT1 can also bind to miR-101 to upregulate PI3K/AKT pathway in epilepsy (203, 204). UCA1 has been found to be differentially hypermethylated in TLE as well (205). By regulating miR-203, UCA1 decreases IL-1β, IL-6, TNF-α, and Cox-2 levels *via* miR-499b-5p in epilepsy (206, 207). In addition, UCA1 might implement the anti-inflammatory function by though AKTmTOR pathway by directly targeting TLR4 (207–209).

### CircRNAs

CircRNA is a closed non-coding RNA which, unlike linear RNA, form a closed-loop structure with a direct ligation of the 3’ and 5’ ends (210). CircRNAs are more stable and abundant than the corresponding linear mRNAs in plasma leading their potentials to be disease biomarkers (211). It also functions as ceRNA to modulate the expression of genes (212). A growing body of studies has uncovered the diverse functions of circRNAs on gene translation and interacting with cellular signaling, such as mTOR pathway (213–218).

Recent studies have assessed the pattern of circRNAs expression in epilepsy (219, 220). For example, circHivep2 can significantly inhibit microglial activation and the expression of inflammatory cytokines in kainic acid (KA)-induced epilepsy (221). It is increasingly recognized that activated astrocytes play a key role in neuronal damages. Similarly, circIgf1r is upregulated in astrocytes in epilepsy and silencing of circIgf1r could play a protective role in neuronal injury by converting reactive astrocytes from Neurotoxic A1 states to A2 phenotype (222). In addition, circRNAs can be cooperate with miRNAs in the development of epilepsy. CircUBQLN, for instance, can directly target mTOR signaling to attenuate oxidative stress in epilepsy *via* reymark(223) the combination with miR-155.

## Conclusion

Neuroinflammation as a major hallmark of seizure plays a key role in the initiation and exacerbation of epilepsy, especially in refractory epilepsy. The of molecular mechanisms underlying epileptic neuroinflammation would largely extend our understanding of epilepsy and benefit the development of targeted therapies. Accumulating evidence has indicated that ncRNAs coordinated with mTOR signaling are essential for regulating the context and course of neuroinflammation and corresponding pathophysiological traits. Moreover, ncRNAs and mTOR have emerged as potential biomarkers, as well as therapeutic targets in the management of epilepsy. However, since only a small fraction of annotated ncRNAs is well characterized, and the interaction of ncRNAs and mTOR signaling in epileptic neuroinflammation requires further studies where functional screening and identification of ncRNAs interoperating in the intricate regulation network would be appreciated.

## Author Contributions

LZ and CZ conceived the review. CZ wrote the draft. All authors contribute to reviewing and editing.

## Conflict of Interest

The authors declare that the research was conducted in the absence of any commercial or financial relationships that could be construed as a potential conflict of interest.

The handling editor WP declared a shared parent affiliation with the authors at the time of review.

## Publisher’s Note

All claims expressed in this article are solely those of the authors and do not necessarily represent those of their affiliated organizations, or those of the publisher, the editors and the reviewers. Any product that may be evaluated in this article, or claim that may be made by its manufacturer, is not guaranteed or endorsed by the publisher.
